# Development of a composite index framework for comprehensive assessment of chemical immobilization performance in tigers (*Panthera tigris*)

**DOI:** 10.3389/fvets.2026.1914647

**Published:** 2026-10-09

**Authors:** Boon Allwin, Anand Rajshekhar Dadke, Kalaignan Pajaniradjou, Sujay Suresh, Sachin Patil, Nitin Tambe, Jayvin Kelaiya, Sivaprakash, Moulidharan, Ratan Gupta

**Affiliations:** Department of Wildlife Health and Management, Greens Zoological Rescue and Rehabilitation Centre, Jamnagar, Gujarat, India

**Keywords:** chemical immobilization, immobilization score, medetomidine–ketamine, recovery quality, tiger

## Abstract

**Introduction:**

Chemical immobilization is an important part of veterinary care in tigers (*Panthera tigris*) as it allows animals to be safely handled for routine health examinations, diagnostic procedures and treatment.

**Methods:**

This study evaluated the clinical performance of a medetomidine (0.04 mg/kg)—ketamine (3 mg/kg) protocol in 40 facility-managed tigers (22 males and 18 females) undergoing routine disease screening and surveillance as part of the Annual Health Checkup. Induction, physiological responses, need for supplemental medication and recovery were assessed using the Immobilization Scoring Index (ISI), Induction Predictability Index (IPI), Protocol Reliability Index (PRI), and Recovery Grading System (RGS). The ISI had a maximum score of 16 and classified immobilization as excellent (13–16), good (9–12), moderate (5–8), or poor (≤4). The IPI was used to assess the consistency of induction, while the PRI indicated how often satisfactory immobilization was achieved with the initial drug dose. Recovery was graded as Grade I (smooth), Grade II (mild ataxia), Grade III (excitable/hyperkinetic), or Grade IV (severe dysphoria). Rectal temperature, respiratory rate, heart rate, capillary refill time, mucous membrane color, and peripheral oxygen saturation were monitored every 10 min during immobilization.

**Results:**

Vital parameters remained within clinically acceptable ranges, and no major anesthetic complications were recorded. Satisfactory immobilization was achieved with the initial dose in 87.5% of tigers, while 12.5% required additional ketamine. The mean ISI score was 13.9 ± 1.1, indicating excellent overall immobilization. The IPI was 5.26%, indicating relatively low variability in induction time within the study population, while the PRI was 87.5%, indicating that most tigers achieved adequate immobilization without supplemental ketamine. Most tigers (92.5%) recovered with Grade I or II responses, indicating smooth to mildly ataxic recovery.

**Discussion:**

Overall, the medetomidine–ketamine protocol provided reliable and clinically satisfactory immobilization in these facility-managed tigers. The proposed scoring systems helped describe different aspects of immobilization in a simple and structured manner. However, further studies are needed to validate these measures before they can be recommended for wider use across different anesthetic protocols or felid species.

## Introduction

Chemical immobilization is an important component of tiger (*Panthera tigris*) management, enabling veterinarians to safely carry out routine health examinations, sample collection, diagnostic procedures, treatment and minor surgical interventions. An effective immobilization protocol should provide predictable induction, adequate and stable anesthesia, minimal need for additional drugs and a smooth recovery (1). However, immobilizing large felids can be challenging. Prolonged induction may increase the risk of excessive physical exertion and injury while the anesthetic procedure itself may be associated with morbidity and mortality (2). Therefore, selecting a reliable and effective immobilization protocol is essential to ensure animal welfare while also maintaining the safety of veterinary and animal-care personnel.

Conventionally, Ketamine–xylazine combinations have been used for the chemical immobilization of Bengal tigers with yohimbine used for reversal (3). More recently, *α*_2_-adrenergic agonists, particularly medetomidine and dexmedetomidine have become widely used in the anesthesia of nondomestic felids because they provide reliable anesthesia, analgesia and muscle relaxation while also allowing pharmacological reversal (4, 5). Among the available protocols, medetomidine–ketamine has been particularly useful for immobilizing nondomestic felids. Studies in facility-managed tigers have reported rapid and effective induction with this combination although additional ketamine may occasionally be required when procedures are prolonged (4). Dexmedetomidine–ketamine protocols have shown similar results, providing effective immobilization with generally stable physiological parameters and few adverse effects (5). Comparable outcomes have also been reported in other wild felid species using medetomidine–ketamine, with atipamezole used for reversal (6). Together, these findings suggest that *α*_2_-agonist-ketamine combinations offer a practical and reversible approach for the chemical immobilization of nondomestic felids.

Although these protocols are generally effective, they are not without potential risks. *α*₂-adrenergic agonists can cause cardiovascular changes, while ketamine may be associated with muscle rigidity, salivation, ataxia, hyperkinesia and excitement during recovery (5). Respiratory and metabolic effects also need to be considered during immobilization. In African lions, medetomidine-based protocols were associated with only minor respiratory and metabolic changes; however, careful monitoring of ventilation, gas exchange, acid–base status, and body temperature was recommended (7). These findings emphasize that maintaining physiological stability is an important component of safe anesthesia.

The overall performance of an immobilization protocol cannot always be judged from a single parameter. For example, a protocol may provide rapid induction but still require supplemental drugs or result in a poor recovery. Appropriate drug dosing is therefore critical. An inadequate dose may lead to delayed or incomplete immobilization, whereas excessive dosing can prolong recovery and increase physiological depression. Previous studies have demonstrated the effectiveness of medetomidine– or dexmedetomidine–ketamine combinations in tigers although individual variation and the occasional need for supplemental ketamine remain important clinical considerations (4, 5). In the present study, medetomidine was administered at 0.04 mg/kg in combination with ketamine at 3 mg/kg, and the protocol was evaluated based on induction consistency, success with the first dose, physiological stability, duration of immobilization, and recovery quality.

Although induction time, immobilization duration, physiological variables, supplemental drug use and recovery are commonly reported in wildlife anesthesia studies these parameters are generally considered separately. Assessing them individually may not provide a complete picture of how well an immobilization protocol performs as a whole and may make comparisons between protocols more difficult. In human medicine, clinical scoring systems have been used to combine multiple clinically relevant observations into a structured assessment. To our knowledge, a composite framework integrating these different aspects of immobilization performance has not been formally described for tiger immobilization.

To address this gap, the present study explored four preliminary measures to organize different aspects of immobilization performance. The Immobilization Scoring Index (ISI) was used to describe overall immobilization quality, Induction Predictability Index (IPI) to describe variation in induction time, Protocol Reliability Index (PRI) to assess successful immobilization with the initial drug dose, and the Recovery Grading System (RGS) to characterize recovery quality. These measures were developed specifically for this study and are intended as descriptive tools rather than validated or universally applicable scoring systems. They provide an initial framework for bringing together clinically relevant observations and describing the overall performance of an immobilization protocol in tigers.

We hypothesized that the medetomidine–ketamine protocol would provide predictable induction, stable physiological parameters, effective immobilization with minimal need for supplemental drugs and smooth recovery. We also expected the proposed preliminary indices to provide a structured way to describe and assess these aspects of immobilization.

Accordingly, this study evaluated the clinical performance of the medetomidine–ketamine protocol in facility-managed tigers, with particular emphasis on induction, physiological stability, requirement for supplemental dosing, duration of immobilization and recovery quality. The study also explored whether the proposed assessment measures could provide a practical and structured way to describe different components of immobilization performance.

## Materials and methods

### Study population

A total of 40 immobilization events were conducted in 40 adult, facility-managed tigers (22 males and 18 females) during the study period. Each tiger underwent a single immobilization event and each event was considered an independent observation for analysis. All animals were in satisfactory body condition (score 4–5) had stable vital parameters and were clinically healthy, with no signs suggestive of infectious disease at the time of assessment. The tigers were examined at the Greens Zoological Rescue and Rehabilitation Centre, Jamnagar, Gujarat, India between April 2022 and April 2024 as part of their Annual Health Assessment. Immobilization was required to safely carry out routine procedures, including physical examination, age estimation using dentition, blood collection, body condition assessment and other diagnostics.

For analysis, immobilization events were grouped according to the time of drug administration into morning (06:00 a.m.–08:00 a.m.), midday (11:00 a.m.–01:00 p.m.) and evening (05:00 p.m.–09:00 p.m.) sessions. Physiological parameters were monitored continuously throughout immobilization, and the values reported in this study represent measurements recorded at 10-min intervals. Heart rate (HR), respiratory rate (RR), rectal temperature (RT) and peripheral oxygen saturation (SpO₂) were monitored regularly. SpO₂ was measured at two sites at the same time: the ear pinna using a multiparameter monitor and the tongue using a pulse oximeter (Edan VE-H100BT™). Capillary refill time (CRT) was assessed on the buccal mucous membrane to provide an additional indication of peripheral perfusion.

### Darting procedure

Drug doses were calculated based on the estimated body weight of each tiger. Body weight was visually estimated by experienced veterinarians using the animal’s overall size, body condition, age, sex and previous clinical records when available. Medetomidine was administered at 0.04 mg/kg in combination with ketamine at 3 mg/kg. Medetomidine hydrochloride (20 mg/mL; compounded formulation supplied by V-Tech, Midrand, South Africa) and ketamine hydrochloride (100 mg/mL; Ketamidor® 10 mL, Richter Pharma, supplied by Girovet, Spain) were administered in their original, undiluted formulations. Remote drug delivery was carried out using a Dan-Inject JM Special JMSPB 16 projector in accordance with the manufacturer’s instructions. Each tiger was darted individually within its enclosure from a distance of 15–20 feet. Depending on the tiger’s position and the safety conditions at the time of darting, the dart was aimed at either the forelimb or hindlimb musculature.

For each immobilization, the dart placement site, delivery status, onset of sedation, and time to adequate immobilization were recorded. Dart delivery was classified as complete, partial, or unsuccessful.

Delivery was considered complete when the dart was empty or the plunger had reached the end of its travel, with no visibly detectable injectate remaining. Partial delivery was recorded when residual drug remained in the dart and the plunger had advanced only partially. In such cases, the attending veterinarian estimated that approximately one-half to two-thirds of the intended volume had been delivered by comparing the residual volume with the original volume loaded. This field-based estimate was not verified using a calibrated volumetric device. Dart bounce-off, inadequate penetration, leakage, or failure of the discharge mechanism was classified as unsuccessful delivery. Because the exact dose received could not be reliably determined after partial or unsuccessful delivery, these events were excluded from the primary analysis. This visual estimation of partial delivery is acknowledged as a limitation of the study.

Only cases with complete drug delivery were included in the primary analysis, as the dose received could not be reliably determined when delivery was partial or unsuccessful. The requirement for supplemental drug was assessed only when the full initial dose had been successfully delivered but the depth of anesthesia remained inadequate. Thus, partial or unsuccessful dart delivery was not considered evidence of inadequate drug effect and was excluded from the assessment of supplemental drug requirement.

### Anesthetic induction and assessment

Following dart administration, the tigers were monitored from a safe distance, with disturbance kept to a minimum during induction. Anesthetic depth was assessed using several clinical signs rather than relying on a single parameter. These included jaw and muscle tone, palpebral and corneal reflexes, ocular globe position, response to external stimulation, pedal withdrawal reflex, ataxia, lateral recumbency and the ear-flick reflex. Unnecessary handling was avoided throughout the induction period.

Adequate immobilization was considered to have been achieved when the tiger showed abolishment of ear-flick reflex. During induction, the ear-flick reflex was assessed remotely from outside the enclosure using a long-handled pole. The concave medial surface of the external pinna (auricle) was gently touched from a safe distance without entering the enclosure. A voluntary ear movement indicated a present reflex, whereas no response indicated its absence. Loss of the ear-flick reflex, together with stable recumbency and absence of other clinically relevant responses, was used as the predefined criterion for approaching the tiger. Abolition of the ear-flick reflex was used as the predefined endpoint for adequate immobilization and safe approach. Ataxia was recorded as the first observable sign of impaired coordination after drug administration, while lateral recumbency was recorded when the animal became fully recumbent and could no longer maintain an upright posture.

For this study, Induction time was defined as the interval from administration of the anesthetic combination until abolition of the ear-flick reflex, which was used as the predefined endpoint for adequate immobilization and safe approach. Immobilization duration was defined as the period from administration of the medetomidine–ketamine combination to administration of reversal (atipamezole). Recovery time was measured from atipamezole administration to the predefined recovery milestones, including return of reflex activity, standing and coordinated ambulation and restoration of normal physiological and behavioral responses. Animals were considered adequately immobilized and safe for handling when they remained unresponsive to standardized stimuli with no voluntary ear flicking, head movement, limb withdrawal or attempts to regain sternal or standing posture.

The tigers were closely monitored throughout anesthesia for any signs of complications. Supplemental oxygen was kept readily available.

### Anesthetic reversal

As a general dosing rule, atipamezole was administered at approximately five times the medetomidine dose on a mg/kg basis. Thus, depending on the medetomidine dose administered and the animal’s estimated body weight, the atipamezole dose was generally 0.2–0.3 mg/kg intramuscularly. Recovery monitoring began immediately after antagonist administration.

### Immobilization performance indices

1. Immobilization Scoring Index (ISI):

The ISI was developed specifically for this study as a preliminary framework to provide an overall description of immobilization quality. Each tiger was assessed across the eight domains presented in Table 1, and the domain scores were added to obtain a total score ranging from 0 to 16. Immobilization was classified as Excellent (13–16), Good (9–12), Moderate (5–8), or Poor (≤4). Higher scores reflected more predictable induction, stable physiological parameters, minimal intervention, and smooth recovery. Physiological stability was judged subjectively by the attending veterinarian using the recorded heart rate, respiratory rate, SpO₂, and rectal temperature. Since objective numerical thresholds had not been established before the study, this domain, and therefore the overall ISI, was considered exploratory rather than a validated measurement tool. Individual ISI scores were summarized as mean ± standard deviation. The domains, scoring thresholds, and classification limits were developed retrospectively on the basis of clinical expectations, published information, and the authors’ practical experience. They were not finalized before data collection or registered in advance. Consequently, the 40 immobilization events served as the developmental dataset, and their classification as “Excellent” represents a descriptive application of the framework rather than independent validation or proof that the categories are universally applicable.

2. Induction Predictability Index (IPI):

**Table 1:** Immobilization Scoring Index (ISI) for evaluation of medetomidine–ketamine immobilization in tigers.

**Domain**	**Parameter**	**Score**	**Criteria**	**Rating basis**
Induction Efficiency	Induction Time	2	<10 min	Rapid induction from drug administration to safe handling
		1	10–15 min	Acceptable but delayed induction
		0	>15 min	Prolonged induction that may increase handling and stress
	Induction Quality	2	Smooth induction without excitation or dysphoria	Calm transition to immobilization with minimal handling risk
		1	Mild excitement, pacing, transient ataxia	Minor excitation that resolves without major intervention
		0	Marked excitation or dysphoria.	Severe excitation, dysphoria, or increased risk of injury
Procedural Adequacy	Supplemental Drug Requirement	2	No supplemental dose required	Immobilization achieved and maintained with the initial dose
		1	One supplemental dose required	Minor inadequate depth or duration requiring one additional dose
		0	Two or more supplemental doses required	Poor or short-lasting immobilization requiring repeated intervention
Physiological Stability (clinician-assigned, semi-quantitative assessment)	Heart Rate [21]	2	50–100 beats/min	Within species-specific physiological limits
		1	40–49 or 101–130 beats/min	Mild bradycardia or tachycardia requiring observation
		0	<40 or >130 beats/min, or clinically important arrhythmia	Marked abnormality with potential clinical significance
	Respiratory Rate and Peripheral Oxygen Saturation (SpO_2_)[5]	2	Respiratory rate 9–32/min and SpO₂ ≥ 95%	Adequate respiratory pattern and oxygenation
		1	Respiratory rate 4–8 or 33–40/min, or SpO₂ 90–94%	Mild respiratory or oxygenation abnormality
		0	Respiratory rate <4 or >40/min, apnea, or SpO₂ <90%	Severe respiratory abnormality or clinically important hypoxemia
	Rectal Temperature [21]	2	37.8–39.4°C	Within physiological limits
		1	36.7–37.7°C or 39.5–40.4°C	Mild hypothermia or hyperthermia requiring monitoring
		0	<36.6°C or >40.5°C	Clinically important hypothermia or hyperthermia
Recovery Characteristics	Recovery Time	2	<25 min	Rapid recovery from antagonism or discontinuation to coordinated movement
		1	25–35 min	Moderately prolonged recovery
		0	>35 min	Prolonged recovery requiring extended monitoring or intervention
	Recovery Quality	2	Smooth recovery with coordinated ambulation	Normal return of coordination without major complications
		1	Mild ataxia or transient excitement	Minor recovery abnormality that resolves without injury
		0	Rough recovery, repeated falls, marked dysphoria, injury risk	Unsafe or clinically concerning recovery
Immobilization Scoring Index (ISI)	Total Score (Maximum = 16)	0–16	Sum of all domain scores	Higher scores indicate more efficient, adequate, physiologically stable, and safer immobilization

These ISI thresholds are practical scoring boundaries, not validated physiological ranges for *Panthera tigris*. They are based on available tiger studies, published *Panthera* data, anesthesia principles, and clinical judgement, and require prospective validation.

IPI was used to describe how consistently the tigers reached adequate induction. It was calculated as the coefficient of variation (CV) of induction time and expressed as a percentage. A lower IPI indicated less variation in induction time between animals and therefore, a more consistent induction response. Since the IPI is based solely on induction time, it was considered a descriptive measure of variability and was not intended to represent an independent or validated measure of overall protocol predictability or reliability.


$$\text{Induction Predictability Index}=(\begin{array}{l} \text{Standard deviation of}\;\\ \text{induction time}/\\ \text{Mean induction time} \end{array})\times 100$$

3. Protocol reliability index (PRI):

The PRI was defined as the percentage of immobilization events in which adequate immobilization was achieved following complete administration of the initial anesthetic dose without supplemental anesthetic administration. PRI is a clinically interpretable representation of first-dose immobilization success.


$$\text{Protocol Reliability Index}=(\begin{array}{l} \text{Number of animals not}\;\\ \text{requiring supplemental dose}/\\ \text{Total number of animals} \end{array})\times 100$$

4. Recovery grading system (RGS):

Recovery quality was assessed using a four-grade system based on the tiger’s coordination, behavior and ability to regain normal posture during recovery from anesthesia. Grade I represented a smooth and coordinated recovery, while Grade II indicated mild ataxia. Grade III described a more excitable or hyperactive recovery, and Grade IV represented severe dysphoria. Lower grades therefore indicated a smoother and more controlled recovery. The RGS was developed specifically for this study as a descriptive tool.

ISI includes recovery as one of several factors used to assess the overall effectiveness of the immobilization protocol. In comparison, RGS specifically evaluates the recovery phase, providing a separate categorical assessment of the quality and smoothness of recovery in each animal.

### Statistical analysis

All analyses were performed using GraphPad Prism™. Normality of the paired differences between estimated and actual body weights was assessed using the Shapiro–Wilk test; as the differences were approximately normally distributed, a paired *t*-test was applied. Data are presented as mean ± SD. Repeated measures of HR, RR, RT and SpO₂ at 10, 20, 30, and 40 min were analyzed using the Friedman test. As no *post hoc* comparisons were performed, only overall time effects were interpreted. Statistical significance was set at *p* < 0.05.

## Results

The mean estimated body weight of the tigers was 133.40 ± 23.85 kg, which was very close to the mean actual body weight of 133.42 ± 22.89 kg. Actual body weight was recorded after immobilization using a calibrated weighing system before completion of the health assessment. Similarly, the estimated doses of medetomidine and ketamine were 5.34 ± 0.95 mg and 400.20 ± 71.55 mg, respectively, compared with 5.34 ± 0.92 mg and 400.28 ± 68.68 mg calculated from the actual body weights (Table 2). A paired *t*-test confirmed that there was no significant difference between estimated and actual body weights (*t*₃₉ = − 0.02, *p* = 0.986).

**Table 2:** Medetomidine–ketamine doses administered in facility-managed tigers (*n* = 40).

**Animal No.**	**Estimated body weight (kg)**	**Estimated dose Medetomidine (mg)**	**Estimated dose Ketamine (mg)**	**Actual body weight (kg)**	**Actual dose Medetomidine (mg)**	**Actual dose Ketamine (mg)**
Mean ± SD	133.40 ± 23.85	5.34 ± 0.95	400.20 ± 71.55	133.42 ± 22.89	5.34 ± 0.92	400.28 ± 68.68

Ataxia was observed at a mean of 2.19 ± 0.27 min after drug administration, followed by lateral recumbency at 2.56 ± 0.17 min. Loss of the ear-flick reflex occurred at 3.50 ± 0.20 min. The mean duration from drug administration to atipamezole administration was 45 ± 3.30 min. After administration of atipamezole, the animals showed restoration of their reflexes within 8.50 ± 1.22 minutes, followed by the return of coordinated ambulation after 24.28 ± 2.5 minutes (Table 3).

**Table 3:** Sequential immobilization responses and anesthesia recovery timeline in facility-managed tigers.

**Immobilization variable**	**Mean time ± SD (min)**
Ataxia	2.19 ± 0.27
Lateral recumbency	2.56 ± 0.17
Loss of ear flick reflex	3.50 ± 0.20
Duration of anesthesia	45 ± 3.30
Time to Reflex Restoration after reversal agent administration	8.50 ± 1.22
Time to Regain Ambulation after reversal agent administration	24.28 ± 2.5

Physiological parameters recorded at 10-minute intervals are summarized in Table 4. HR, RR and RT showed significant variation over the observation period (*P* < 0.001 for all three parameters). HR gradually decreased from 55 ± 4 beats/min at 10 min to 51 ± 2 beats/min at 40 min. Respiratory rate remained relatively stable, ranging from 20 ± 2 to 19 ± 1 breaths/min. RT showed a mild decline from 38.6 ± 0.3°C at 10 min to 38.2 ± 0.2°C at 40 min. SpO₂ remained stable throughout the procedure, with no significant difference between time points (*P* = 0.139). CRT remained below 2 sec and the mucous membranes remained pale pink throughout the recorded period.

**Table 4:** Time-dependent physiological response pattern during post-induction immobilization in facility-managed tigers.

**Vital signs**	**10th minute**	**20th minute**	**30th minute**	**40th minute**	**Physiological Range [5,21]**
Rectal temperature (°C)	38.6 ± 0.3	38.6 ± 0.2	38.6 ± 0.2	38.2 ± 0.2	37.8 - 39.4
Respiratory rate (breaths/min)	20 ± 2	22 ± 1	20 ± 1	19 ± 1	9-32
Heart rate (beats/min)	55 ± 4	55± 4	52 ± 2	51 ± 2	50-100
Capillary refill time (CRT)	< 2 sec	< 2 sec	< 2 sec	< 2 sec	NA
Color of mucous membrane	Pale pink	Pale pink	Pale pink	Pale pink	NA
Peripheral Oxygen Saturation **(**SpO2)	98 ± 1 %	99 ± 1 %	99 ± 1 %	99 ± 1%	NA

The mean ISI was 13.9 ± 1.1 out of a maximum score of 16 and fell within the proposed excellent category of the exploratory scoring framework. The IPI was 5.26%, while the PRI was 87.5%. Supplemental ketamine was not required in 35 of the 40 immobilization events, whereas supplementation was required in only five cases.

Recovery was graded as Grade I in 67.5% of animals (27/40), Grade II in 25.0% (10/40), Grade III in 5.0% (2/40), and Grade IV in 2.5% (1/40). Overall, 37 of the 40 tigers (92.5%) had Grade I or II recovery, indicating predominantly smooth and satisfactory recovery following reversal. None of the tigers required supplemental oxygen or emergency intervention during the study period.

Mean ISI scores were comparable across the different times of day, with values of 13.90 ± 1.21 in the morning group (*n* = 20), 14.00 ± 0.89 at midday (*n* = 6), and 13.93 ± 1.21 in the evening group (*n* = 14). One-way ANOVA showed no significant difference among the groups (*F* = 0.015, *p* = 0.985). Similarly, ISI scores did not differ between animals darted in the forelimb (13.90 ± 1.20; *n* = 10) and those darted in the hindlimb (13.93 ± 1.18; *n* = 30), with no significant difference between the two groups (*t* = 0.08, *p* = 0.94).

## Discussion

Chemical immobilization is an important part of routine veterinary management in facility-managed tigers allowing animals to be safely examined, sampled, treated and handled when required. In the present study, the medetomidine–ketamine protocol provided a relatively consistent pattern of induction, adequate immobilization duration, limited requirement for supplemental ketamine and generally favorable recovery across the 40 tigers evaluated. The immobilization protocol provided a rapid and consistent onset of effect, with most animals achieving satisfactory immobilization without additional dosing. The overall immobilization quality and generally smooth recovery indicate that the protocol was effective and well tolerated. Taken together, these findings suggest that the protocol performed consistently and was practically reliable under the conditions of the present study.

These findings suggest that experienced visual estimation of body weight can be a useful practical approach for drug-dose planning when direct weighing is not feasible or reliable previous weight records are unavailable. Therefore, visual estimation may be useful in field situations, but should not be considered a substitute for direct body-weight measurement. Although numerous anesthetic protocols have been described for tigers, assessment of immobilization success has traditionally relied upon isolated outcome measures such as induction time, immobilization duration, physiological responses, and recovery characteristics (3–5, 8).

The induction characteristics observed in this study are broadly comparable with previous reports of *α*₂-agonist-ketamine combinations in tigers and other nondomestic felids (4–7). Medetomidine provides sedation, analgesia and muscle relaxation, while ketamine contributes dissociative anesthesia, making the combination useful for short-duration procedures in large felids. Effective immobilization using medetomidine–ketamine has previously been reported in captive tigers (4) while dexmedetomidine–ketamine has also been evaluated successfully in this species (5). Similar *α*₂-agonist-based protocols have been reported in other large felids, including clouded leopards and African lions (6, 7). Direct comparison between studies should nevertheless be made cautiously because drug doses, routes and methods of administration, animal characteristics, monitoring methods, and duration of procedures may differ considerably.

The findings are generally consistent with previous reports describing alpha-2 agonist– dissociative anesthetic combinations in tigers and other large felids. Early studies utilizing ketamine–xylazine combinations demonstrated acceptable restraint but often reported variable induction and recovery characteristics. The introduction of medetomidine represented a significant advancement owing to its greater alpha-2 receptor selectivity, enhanced sedative potency, improved muscle relaxation, absence of urination and complete pharmacological reversibility (9, 10). Comparable findings reported in tigers, clouded leopards and lions support the utility of alpha-2 agonist–based protocols across felid species.

The synergistic interaction between these agents improves immobilization quality while reducing undesirable effects associated with administration of either drug alone (11, 12). The time of anesthesia administration did not significantly influence the ISI. Mean scores were similar among morning, midday, and evening immobilizations. Similarly, no significant difference in ISI was observed between animals darted in the forelimb and those darted in the hindlimb. These findings suggest that, under the conditions of the present study, time of day was not associated with any measurable difference in overall immobilization performance, indicating that photoperiod-related or circadian variations had little apparent influence. The selected darting site also did not significantly affect immobilization performance. However, the number of animals in some subgroups was relatively small, particularly in the midday group and the forelimb darting group. These findings should therefore be interpreted cautiously and should not be taken as evidence that time of day or dart placement can never influence immobilization outcomes.

The physiological changes recorded during immobilization were generally consistent with the expected effects of medetomidine. Heart rate showed a gradual decline during the observation period, which is compatible with the cardiovascular effects of *α*₂-adrenergic agonists, including peripheral vasoconstriction and reflex bradycardia (4, 7, 13). Respiratory rate showed relatively small fluctuations, while rectal temperature decreased toward the later observation period. These changes highlight the importance of regular monitoring of cardiovascular and thermal status during immobilization, particularly when procedures are prolonged. SpO₂ remained relatively stable throughout the monitored period. However, because capnography and arterial blood gas analysis were not performed, the present study provides limited information regarding ventilation and carbon dioxide retention. Future studies incorporating these measurements would allow a more complete assessment of the respiratory effects of the protocol.

The mean ISI score was 13.9 ± 1.1, with a maximum possible score of 16, placing the mean score within the predefined excellent category of the proposed scoring system. The purpose of the ISI was to bring together several aspects of an immobilization event, including induction, procedural adequacy, physiological stability, and recovery, rather than relying on a single parameter. In this respect, the ISI provides a broader description of immobilization performance than induction time or immobilization duration alone. The domains, thresholds, and equal weighting were based on clinical judgement, published literature, and the authors’ experience. They were not formally validated or adapted from an established tiger-specific scoring system. Thresholds were set before final ISI scores and outcome classifications and reflected practical, physiologically plausible limits—not normal reference ranges.

PRI and IPI provide additional information that is not captured by the ISI alone. A PRI of 87.5% meant that 35 of the 40 immobilization events were completed without the need for supplemental ketamine. This finding has important operational implications because repeat darting may increase handling duration, preexcitation, physiological stress, risk of hyperthermia, and potential injury to both animals and personnel (14, 15). The IPI was 5.26%, representing the coefficient of variation in induction time. Rather than assigning a fixed clinical category to this value, the IPI provides a quantitative measure of the variability in induction time within the study population. The relatively consistent induction endpoints observed in the present study suggest that the protocol produced a fairly predictable onset under the conditions evaluated. However, because there are currently no established IPI reference values for tiger immobilization, the clinical significance of a particular IPI value will require assessment in larger and independent populations.

Recovery was another important component of the overall immobilization assessment. In the present study, 67.5% of animals had Grade I recovery and 25.0% had Grade II recovery, while Grade III and Grade IV recoveries occurred in 5.0 and 2.5% of animals, respectively. Thus, 92.5% of animals were classified as Grade I or II. The predominance of these grades indicates that most animals showed smooth to mildly ataxic recovery following reversal. Atipamezole provides pharmacological reversal of medetomidine-induced sedation, which likely contributed to the relatively predictable recovery observed in this study.

The proposed ISI, PRI, IPI, and RGS indices were developed on principles similar to other clinical scoring systems that combine multiple variables to allow more consistent assessment of outcomes. Widely adopted examples include the Glasgow Coma Scale, APACHE II, and SOFA scoring systems, all of which integrate multiple variables into clinically meaningful frameworks (16–18). Although the indices proposed in the present study serve a fundamentally different purpose, they are based on the same principle that overall clinical performance is often best represented through integration of multiple outcome measures rather than reliance on a single variable.

One of the main aims of the present study was to move beyond reporting individual anesthesia variables and explore whether several clinically relevant outcomes could be brought together into a structured assessment framework. ISI, IPI, PRI and RGS were designed to describe different components of an immobilization event. The ISI provides an overall composite score, the IPI describes variation in induction time, the PRI describes first-dose success, and the RGS describes recovery quality. Together, these measures provide a more structured way of reporting immobilization outcomes. However, these indices were developed specifically for this study and should currently be regarded as preliminary tools. Their performance in other institutions, different tiger populations, other anesthetic protocols, or other felid species remains unknown.

Nevertheless, to our knowledge, this is an initial attempt to develop a composite framework integrating multiple components of immobilization performance in a nondomestic felid integrating induction quality, procedural reliability, physiological stability, and recovery outcomes into a unified assessment model. Further validation across institutions and species is needed before these indices can be recommended for wider use. A persistent challenge within wildlife anesthesia research is the lack of standardized outcome measures capable of capturing the overall success of an immobilization event. Consequently, comparison among studies, institutions, and immobilization protocols is often complicated by differences in reporting methodology and outcome selection. Similar challenges have historically been encountered in human medicine and critical care, where composite scoring systems were developed to improve standardization, reproducibility, and objective comparison of clinical outcomes (16–18).

The development of a composite scoring system also requires careful consideration of how individual parameters are selected and weighted. In the present ISI, equal weighting was used because there is currently insufficient evidence to suggest that one component should contribute more than another to overall immobilization performance in tigers. The scoring thresholds were based on clinical expectations, available literature and the author’s practical experience rather than being derived from a formal validation process. For this reason, the present scoring system should be considered the developmental stage of a potential assessment framework. Formal evaluation of content validity, construct validity, inter-observer reliability, repeatability, responsiveness, and external applicability will be necessary before the indices can be considered validated measurement tools (19, 20).

Several limitations should be considered when interpreting the findings. The study was conducted at a single zoological institution and included 40 facility-managed adult tigers, which may limit the extent to which the findings can be generalized to other institutions or tiger populations. The study also did not include a comparator anesthetic protocol and therefore it cannot determine whether medetomidine–ketamine performs better than other commonly used combinations. Advanced cardiopulmonary monitoring, including capnography and arterial blood gas analysis was not performed limiting the assessment of ventilation and gas exchange. In addition, the anesthetic doses were initially calculated using estimated body weight rather than measured body weight. Although the estimated and actual weights were closely similar in this population, individual differences may still have occurred. Finally, ISI, IPI, PRI, and RGS were developed and evaluated within the same study population and have not yet undergone external validation. The routine clinical nature of the immobilizations also meant that factors such as animal temperament, enclosure conditions, dart placement, and procedural requirements could not be fully standardized.

Future studies should therefore focus on prospective and multicenter evaluation of the proposed scoring framework using larger populations. Assessment by independent observers would help determine inter-observer agreement and repeatability, while comparison with other anesthetic protocols could establish whether the indices are able to detect meaningful differences in immobilization performance. Incorporating capnography, arterial blood gas analysis and other advanced monitoring techniques would also strengthen the assessment of physiological stability. Further work should determine whether the current ISI domains and thresholds require modification or differential weighting based on data from larger populations.

In conclusion, the medetomidine–ketamine protocol provided predictable induction, an adequate period of immobilization, stable monitored physiological parameters, and predominantly favorable recovery in the facility-managed adult tigers evaluated in this study. The ISI, IPI, PRI, and RGS provided a structured approach for describing different aspects of immobilization performance. Although developed from data in *Panthera tigris*, the framework has potential translational relevance to other *Panthera* species, supported by their close phylogenetic relationship, conserved felid physiology and broadly comparable responses to commonly employed immobilization agents. However, species- and protocol-specific validation remains essential before broader application, particularly for the numerical thresholds, which should not be considered definitive clinical reference values without further validation. The findings therefore provide an initial framework for further investigation rather than establishing universal standards for tiger immobilization. Prospective, multicenter studies involving larger populations and independent assessment will be required to determine the reliability, validity, and broader clinical applicability of these indices.

## Data Availability

The raw data supporting the conclusions of this article will be made available by the authors, without undue reservation.
